# Replication-dependent histone isoforms: a new source of complexity in chromatin structure and function

**DOI:** 10.1093/nar/gky831

**Published:** 2018-09-13

**Authors:** Rajbir Singh, Emily Bassett, Arnab Chakravarti, Mark R Parthun

**Affiliations:** 1 Department of Radiation Oncology, The Ohio State University, Columbus, OH 43210, USA; 2 Department of Biological Chemistry and Pharmacology, The Ohio State University, Columbus, OH 43210, USA

*Nucleic Acids Research*, gky768, https://doi.org/10.1093/nar/gky768

**Figure 2.**
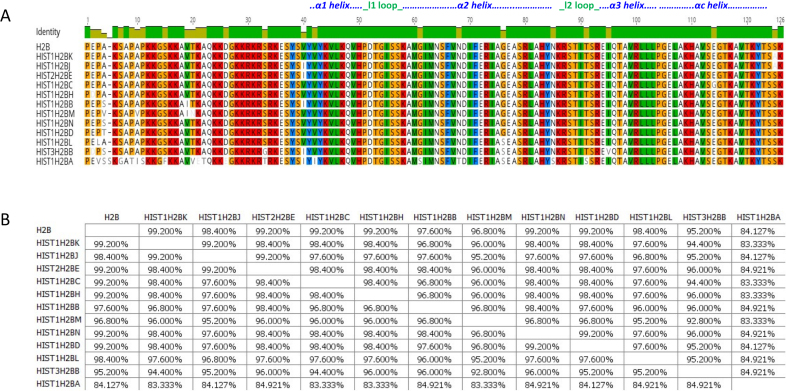
Comparison of H2B isoforms. (**A**) Sequence alignment of H2B isoforms. Residues that are different among the isoforms are shown. (**B**) Percentage similarity among the H2B isoforms based on their primary sequence. Images were created using the Geneious software.

**Figure 3.**
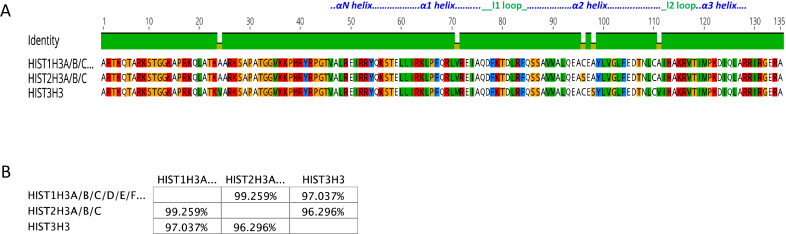
Comparison of H3 isoforms. (**A**) Sequence alignment of H3 isoforms. Residues that are different among the isoforms are shown. (**B**) Percentage similarity among the H3 isoforms based on their primary sequence. Images were created using the Geneious software.

**Figure 4.**
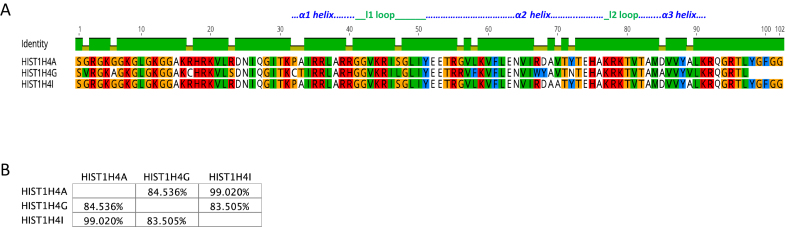
Comparison of H4 isoforms. (**A**) Sequence alignment of H4 isoforms. Residues that are different among the isoforms are shown. (**B**) Percentage similarity among the H4 isoforms based on their primary sequence. Images were created using the Geneious software.

In Figures 2A, 3A and 4A, the schematic diagrams at the top of the figures showing the secondary structure of the histones are mis-aligned with the sequences below them. Correct figures are provided below and have been updated in the published article.

